# Translational demand is not a major source of plasmid-associated fitness costs

**DOI:** 10.1098/rstb.2020.0463

**Published:** 2022-01-17

**Authors:** Jerónimo Rodríguez-Beltrán, Ricardo León-Sampedro, Paula Ramiro-Martínez, Carmen de la Vega, Fernando Baquero, Bruce R. Levin, Álvaro San Millán

**Affiliations:** ^1^ Department of Microbiology, Ramón y Cajal Institute for Health Research (IRYCIS), Ramón y Cajal University Hospital, Madrid, Spain; ^2^ Centro de Investigación Biológica en Red, Epidemiología y Salud Pública (CIBERESP), Instituto de Salud Carlos III, Madrid, Spain; ^3^ Department of Biology, Emory University, Atlanta, GA, USA; ^4^ Antibiotic Resistance Center, Emory University, Atlanta, GA, USA; ^5^ Department of Microbial Biotechnology, Centro Nacional de Biotecnología–CSIC, 28049 Madrid, Spain

**Keywords:** plasmid, fitness cost, translational demand, codon usage, ribosome, horizontal gene transfer

## Abstract

Plasmids are key drivers of bacterial evolution because they are crucial agents for the horizontal transfer of adaptive traits, such as antibiotic resistance. Most plasmids entail a metabolic burden that reduces the fitness of their host if there is no selection for plasmid-encoded genes. It has been hypothesized that the translational demand imposed by plasmid-encoded genes is a major mechanism driving the fitness cost of plasmids. Plasmid-encoded genes typically present a different codon usage from host chromosomal genes. As a consequence, the translation of plasmid-encoded genes might sequestrate ribosomes on plasmid transcripts, overwhelming the translation machinery of the cell. However, the pervasiveness and origins of the translation-derived costs of plasmids are yet to be assessed. Here, we systematically altered translation efficiency in the host cell to disentangle the fitness effects produced by six natural antibiotic resistance plasmids. We show that limiting translation efficiency either by reducing the number of available ribosomes or their processivity does not increase plasmid costs. Overall, our results suggest that ribosomal paucity is not a major contributor to plasmid fitness costs.

This article is part of the theme issue ‘The secret lives of microbial mobile genetic elements’.

## 1. Background

Horizontal gene transfer (HGT) shapes bacterial evolution, allowing bacteria to expand to new ecological niches and thrive in a plethora of different environmental conditions [1]. Among the drivers of HGT, plasmids stand out as one of the most important vehicles for genetic exchange. Plasmids are self-replicative circular DNA fragments that typically carry important adaptive bacterial traits such as antibiotic resistance, virulence and metabolic genes [2]. However, plasmids tend to cause a burden to bacterial physiology and, in the absence of selection for plasmid-encoded traits, plasmid carriage often translates into a reduction in bacterial fitness [3]. The fitness cost of plasmids is one of the major limitations to their spread and persistence in bacterial populations and challenges our understanding of their existence conditions (a conundrum known as the ‘plasmid paradox’) [4].

Over recent years, several works have shed light on the molecular mechanisms that underlie the cost of plasmids (reviewed in [3,5]). These studies showed that the sources of plasmid costs are diverse, and arise from the impact of plasmid biology on multiple cellular processes. During their life cycle, plasmids might, for example, sequestrate the bacterial replication and gene expression machinery, alter the transcriptional profile of their host and drive the expression of dozens of foreign proteins that can negatively interact with host cellular networks [3,5–9].

The universality of these potential sources of plasmid costs is a matter of debate, yet it is generally accepted that the perturbation produced by plasmids (and other forms of HGT) on the translational machinery of the cell leads to a fitness cost [8,10–13]. This perturbation stems from the fact that plasmids and chromosomes usually present different GC content, and consequently, plasmid genes typically show a different codon usage bias from the host chromosome [14]. This discrepancy creates an imbalance between charged tRNA abundance in the cellular pool and the tRNAs specified in the codons of plasmid genes. Therefore, the abundance of some tRNAs becomes suboptimal, leading to the sequestration of rare tRNAs and ribosomes in foreign transcripts, and slowing down the elongation of nascent proteins. As a result, the overall translation efficiency of the cell is reduced and the host fitness decreases. In agreement with this idea, computational studies have shown that codon usage similarity between host and transferred genes is a major determinant of HGT compatibility, and hence organisms with similar tRNA pools tend to exchange more genes [11,13]. Similarly, experimental evidence indicates that translation of highly expressed proteins with suboptimal codon usage causes growth defects in *Escherichia coli* [10,15]. This, and other evidence [16,17], suggests translational demand as a key barrier for the spread of plasmids.

In this work, we sought to explore the importance and pervasiveness of the translational demand of plasmid-borne genes in the fitness effects associated with plasmid acquisition. We hypothesized that if translational demand is a major source of plasmid costs, cells with reduced translational efficiency should show increased plasmid-associated costs. In other words, plasmid carriage and reduced translational capability should show negative (synergistic) epistasis for fitness. This hypothesis is based on the following three premises: (i) the translation machinery of wild-type *E. coli* is highly optimized to maximize growth rate in different environments and metabolic states [18,19]; (ii) exogenous genes compete with housekeeping genes for the translational apparatus, and thus the production of plasmid proteins diverts resources from the synthesis of essential proteins [20,21]; and (iii) bacterial growth rates are proportional to the available ribosomes and their peptide chain elongation rate [22]. To test this hypothesis, we took advantage of a collection of *E. coli* mutant strains that either have reduced ribosomal availability or processivity, and experimentally measured plasmid fitness costs for six diverse clinically relevant plasmids. Our results suggest that the translational demand of plasmid genes is not enough to account for their cost, suggesting that additional mechanisms might explain why plasmids tend to be costly in the absence of direct selection.

## 2. Results

### (a) Fitness effects of six natural plasmids

**Figure 1.**
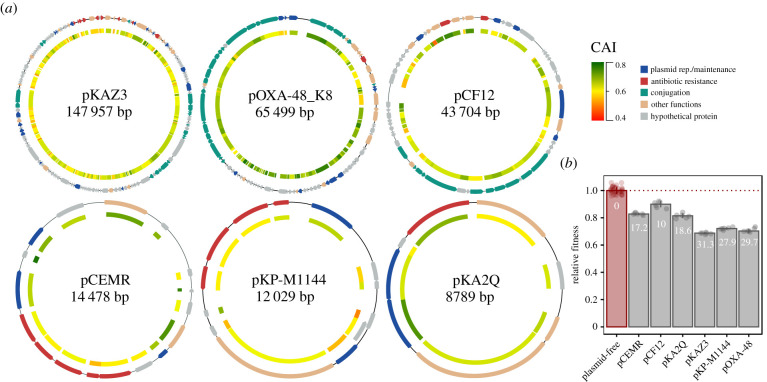
Plasmids and their fitness effects. (*a*) Diagram depicting the six plasmids used in this study, showing the open reading frames (ORFs) as arrows (outer track), with the arrowhead indicating the direction of transcription, and coloured according to their function (see legend). The inner track represents the codon adaptation index (CAI [23]) for each ORF (see legend for colour reference). pOXA-48_K8 is a pOXA-48-like plasmid, and for simplicity, we refer to this plasmid as pOXA-48 throughout the study. (*b*) Fitness effects associated with plasmid acquisition in *E. coli* MG1655. Each bar corresponds to the median value of six replicates (36 for MG1655) with each point representing an independent biological replicate. Numbers within each bar show the cost of each plasmid (% reduction in fitness relative to plasmid-free MG1655).

To test the role of the translational demand of plasmids in their fitness costs we selected six diverse clinically relevant plasmids. These plasmids belonged to different incompatibility groups, carried diverse antibiotic resistance genes, and varied largely in GC content (46–61%), size (8–147 kb) and copy number (figure 1*a*; electronic supplementary material, table S1 and figure S1). These plasmids were originally isolated from various species of the *Enterobacteriaceae* family including *Klebsiella pneumoniae*, *Citrobacter freundii*, *Kluyvera ascorbata* and *Enterobacter cloacae*, and fell in different categories according to their mobility (conjugative, mobilizable and non-transmissible plasmids) [24].

We independently introduced these plasmids either by transformation (pKA2Q, pKP-M1144 and pCEMR) or conjugation (pOXA-48, pCF12 and pKAZ3) in *E. coli* MG1655 and quantified the plasmid fitness effects by performing head-to-head competition experiments against an MG1655 derivative carrying arabinose-inducible chromosomal copy of green fluorescent protein (*gfp*) (MG1655::*gfp*) as a common competitor. Importantly, competition assays integrate all parameters from the growth curve (lag phase, maximum growth rate and carrying capacity) and are extremely sensitive [25], allowing us to obtain precise estimations of the relative fitness of each plasmid-carrying strain. We verified that no significant plasmid loss or transfer (less than 1%) occurred during the competition by plating on appropriate antibiotics. The results showed that all plasmids produced significant costs that ranged from a 10 to a 30% reduction in relative fitness (one way ANOVA effect of plasmid *F* = 429.56, d.f. = 6, *p* = 2.79 × 10^−50^ and Tukey adjusted *p* < 1.49 × 10^−12^ in all cases; figure 1*b*). There were no significant correlations between plasmid size, GC content or average codon bias (measured as codon adaptation index, CAI [23], which measures codon usage similarity between plasmids and highly expressed MG1655 chromosomal genes) and plasmid-associated costs (electronic supplementary material, figure S2, Spearman rank correlation *p* > 0.35).

### (b) Limiting ribosomal processivity does not increase plasmid-associated costs

**Figure 2.**
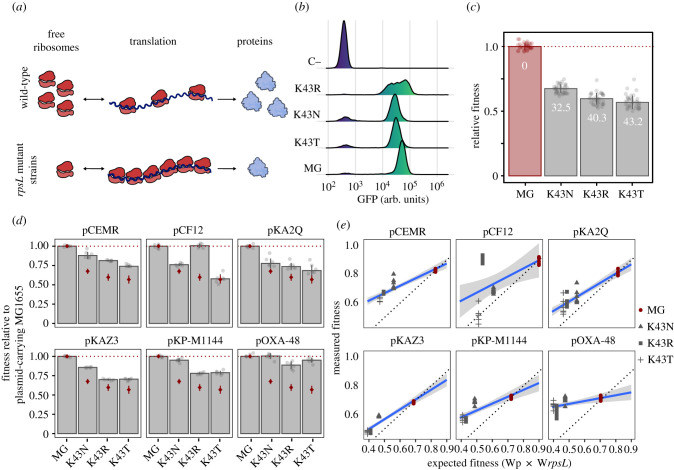
Reducing ribosomal elongation rates does not increase plasmid costs. (*a*) Slow ribosomal elongation rates caused by *rpsL* mutations lead to an increased number of transcript-bound ribosomes, thereby depleting the pool of available ribosomes and consequently reducing translation rate. This is illustrated by comparing translation in wild-type cells (above) and *rpsL* mutants (below). Note that the total number of ribosomes is conserved between both panels. (*b*) After 2 h of induction with 0.1% l-arabinose, strains with *rpsL* mutations show lower fluorescence levels (in arbitrary units; arb. units) than MG1655 (MG) as measured by flow cytometry, indicating a reduced translation capability. For reference, an uninduced MG1655 control (C−) is included. (*c*) Fitness of strains carrying *rpsL* mutations relative to MG1655. Each bar corresponds to the median value of 36 replicates, with each point representing an independent biological replicate. Error bars depict standard deviation. Numbers within each bar show the cost of each mutation (% reduction in relative fitness relative to plasmid-free MG1655). (*d*) Fitness of the plasmid–*rpsL* mutant strain combinations relative to the fitness of plasmid-carrying MG1655. The height of the bar represents the median fitness of each plasmid–*rpsL* mutant strain combination relative to MG1655 carrying each of the plasmids. Red diamonds represent median fitness values of *rpsL* mutants relative to MG1655 (as in figure 2*c*). Therefore, bars below their respective diamond indicate greater plasmid costs (i.e. lower fitness), whereas those above the diamond show lower plasmid-associated costs than those found in MG1655. Error bars represent standard deviation and each point represents an independent biological replicate (*n* = 6). (*e*) Relationship between expected fitness calculated as the product of the fitness effects of each plasmid (Wp) and mutation (W*rpsL*) separately and the fitness measured for each plasmid–mutant combination in competition experiments. Data points above the grey dotted line indicate positive epistasis, whereas those points below show negative epistasis (i.e. increased plasmid-associated costs). Genotypes are depicted using different symbols (see legend). The blue line shows linear regression of the data with 95% confidence intervals (grey shading).

It is well established that a reduction in ribosomal processivity leads to the sequestration of ribosomes on mRNA, depleting available ribosomes from the cellular pool and leading to a reduced cellular translation efficiency (figure 2*a*) [8,10]. We argued that if a plasmid-associated increase in translational demand is responsible for plasmid fitness costs, these costs should increase in cells with reduced ribosomal elongation rates. To test this possibility, we took advantage of the fact that mutations in the *rpsL* gene encoding the 30S ribosomal protein S12 confer resistance to the aminoglycoside antibiotic streptomycin, but at the expense of negatively affecting ribosomal elongation rates [26,27]. We selected three *E. coli* MG1655 spontaneous streptomycin-resistant mutants bearing different mutations in the *rpsL* gene (mutations K43N, K43R and K43T), which have been shown to reduce protein elongation rates [26,27]. To obtain a proxy of protein production rate, we transformed each strain with the plasmid pBGT-1, which is a medium copy plasmid (approx. 15 copies cell^−1^) that carries the *gfp* gene under the control of the P_BAD_ promoter [28]. After overnight growth, we induced GFP production for 2 h with 0.1% l-arabinose and measured GFP fluorescence using a flow cytometer. Importantly, GFP fluorescence is known to strongly correlate with GFP protein levels, indicating that fluorescence can be reliably used as a proxy for protein abundance [10]. In agreement with previous reports [26,27], *rpsL* mutants showed reduced protein production rates (figure 2*b*), which translated into strong fitness defects in comparison to the wild-type MG1655 strain (one way ANOVA effect of plasmid *F* = 889.40, d.f. = 3, *p* = 6.40 × 10^−91^ and Tukey adjusted *p* < 1.26 × 10^−14^ in all cases; figure 2*c*).

We independently introduced the six plasmids into the three *rpsL* mutants and measured their fitness relative to their plasmid-free MG1655 parental strain. We found that in the *rpsL* mutants most plasmids produced no significant costs, but instead usually provided benefits (typically ranging from 4 to 7% fitness advantage, but as high as 51% for pCF12-K43R; figure 2*d*). Only 3 out of the 18 plasmid–*rpsL* mutant combinations showed significant costs compared to their respective plasmid-free parental strain (all mutants carrying plasmid pKAZ3; Tukey adjusted *p* < 3.15 × 10^−4^). Overall, most of the *rpsL* plasmid–mutant combinations showed smaller costs than those found for MG1655 (Tukey adjusted *p* < 7.76 × 10^−3^ in all cases), with the only exception being the pCF12 plasmid in the *rpsL* K43T mutant (Tukey adjusted *p* = 0.92; figure 2*d* and electronic supplementary material, figure S3).

To better understand this result, we calculated the expected fitness for each plasmid–mutant combination as the product of the effects of each plasmid and mutation separately (relative to plasmid-free MG1655; see Methods). We found that the observed fitness of 17 out of the 18 plasmid-carrying *rpsL* mutants is higher than expected by the multiplicative null model (figure 2*e*), confirming the previous observation that *rpsL* mutations and plasmid carriage show positive epistasis [29] (ANOVA effect of plasmid × genotype interaction *F* = 38.79, d.f. = 18, *p* < 10^−62^ and Tukey adjusted *p* < 9.01 × 10^−4^ in all cases except pCF12-*rpsL* K43T, which shows no significant epistasis; electronic supplementary material, figure S4). Of note, four of these combinations showed sign epistasis, in which the strain carrying both resistance determinants is fitter than the strain carrying only the mutation or only the plasmid (Tukey adjusted *p* < 3.34 × 10^−4^ for pCEMR-K43N, pCEMR-K43R, pCF12-K43R and pOXA-48-K34T). Together, these results highlight that artificially reducing ribosomal elongation rates does not increase (and instead tends to decrease) plasmid costs.

### (c) Limiting ribosomal availability does not increase plasmid costs

**Figure 3.**
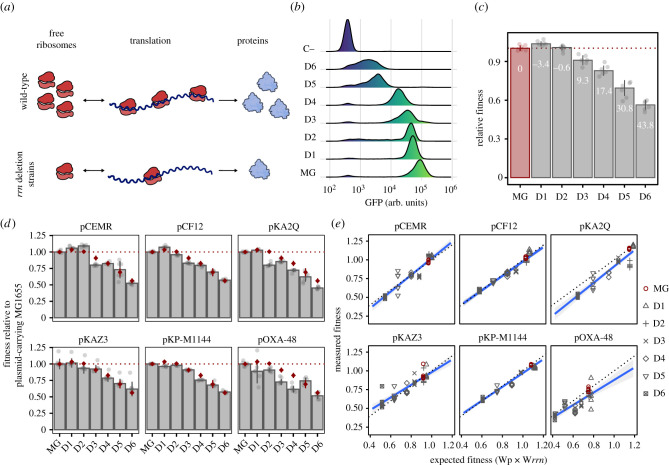
Reducing ribosomal availability does not increase plasmid costs. (*a*) Reduced ribosomal availability depletes the pool of available ribosomes, consequently reducing translation rate. This is illustrated by comparing translation in wild-type cells (above) and *rrn* deletion strains (below). (*b*) After 2 h of induction with 0.1% l-arabinose, strains with reduced ribosomal availability show lower fluorescence levels (in arbitrary units; arb. units) than MG1655 (MG) as measured by flow cytometry, indicating a reduced translation capability. For reference, an uninduced MG1655 control (C−) is included. (*c*) Fitness of strains carrying *rrn* deletions relative to MG1655. Each bar corresponds to the median value of six replicates, with each point representing an independent biological replicate. Error bars depict standard deviation. Numbers within each bar show the cost of each deletion (% reduction in relative fitness relative to plasmid-free MG1655). (*d*) Fitness of the plasmid–*rrn* deletion strain combinations relative to the fitness of plasmid-carrying MG1655. The height of the bar represents the median fitness of each plasmid–*rrn* deletion strain combination relative to MG1655 carrying each of the plasmids. Red diamonds represent median fitness values of *rrn* deletion strains relative to MG1655 (as in figure 3*c*). Therefore, bars below their respective diamond indicate greater plasmid costs (i.e. lower fitness), whereas those above the diamond show lower plasmid-associated costs than those found in MG1655. Error bars represent standard deviation and each point represents an independent biological replicate (*n* = 6). (*e*) Relationship between expected fitness calculated as the product of the fitness effects of each plasmid (Wp) and mutation (W*rrn*) separately and the fitness measured for each plasmid–mutant combination in competition experiments. Data points above the grey dotted lines indicate positive epistasis, whereas those below show negative epistasis (i.e. increased plasmid-associated costs). Genotypes are depicted using different symbols (see legend). The blue lines show linear regression of the data with 95% confidence intervals (grey shading).

We next decided to test our hypothesis using a collection of strains with reduced ribosomal availability (figure 3*a*). As biosynthesis of new ribosomes is primarily determined by ribosomal RNA (rRNA) transcription, deletion of *rrn*A (*rrn*) operons leads to a reduced ribosomal availability [30,31]. We used a set of MG1655 mutant strains carrying sequential deletions from one to six out of the seven *E. coli rrn* operons [31] (electronic supplementary material, figure S5). These strains, denoted D1–D6 to indicate the number of *rrn* operons deleted, show reduced levels of functional ribosomes [32], and thus a reduced translational capability (figure 3*b*).

We measured the fitness effects caused by the deletions of the *rrn* operons and, in agreement with previous reports [31,32], we observed significant costs associated with the deletion of more than 3 *rrn* operons (ANOVA effect of plasmid *F* = 152.10, d.f. = 7; *p* = 9.11 × 10^−27^ and Tukey adjusted *p* < 7.05 × 10^−4^; figure 3*c*). Deletion of 1 or 2 *rrn* operons did not produce any measurable fitness defect, likely because *rrn* expression is controlled through feedback loops that increase the expression of the remaining operons to maintain appropriate ribosome levels [22,33]. We introduced the six plasmids in the *rrn* deletion strains and the parental MG1655 to give rise to 36 plasmid–*rrn* mutant combinations and six MG1655/plasmid controls and calculated plasmid costs relative to their plasmid-free parental strain through competition assays.

Although some plasmid–*rrn* deletion strain combinations showed significantly higher plasmid costs than those observed in MG1655 (11/36 plasmid–*rrn* deletion combinations, Tukey adjusted *p* < 0.04 after significant ANOVA; figure 3*d* and electronic supplementary material, figure S6), in the majority of cases deletion of *rrn* operons did not significantly increase plasmid costs (25/36 plasmid–*rrn* deletion combinations; Tukey adjusted *p* > 0.07; figure 3*d* and electronic supplementary material, figure S6). Next, we calculated the expected fitness of each plasmid–*rrn* deletion combination using the multiplicative model explained above and found that there were only 9/36 instances of significant negative epistasis (Tukey adjusted *p* < 0.05; figure 3*e* and electronic supplementary material, figure S7). Moreover, we did not find a significant correlation between the number of *rrn* operons deleted and plasmid costs (Spearman rank correlation *p* = 0.09, *ρ* = −0.10 for all data together, and *p* > 0.17 for each plasmid individually; electronic supplementary material, figure S8). Taken together, these results suggest that reducing ribosomal availability through deletion of the *rrn* operons does not increase plasmid costs, as cells with fewer *rrn* operons available do not typically show greater plasmid-associated fitness costs.

## 3. Discussion

In this work, we used a diverse set of natural plasmids conferring antibiotic resistance to investigate the role of translational demand on plasmid fitness effects. We used two sets of strains that presented either reduced ribosomal elongation rates (*rpsL* mutants) or availability (*rrn* deletion strains) and found that, although specific plasmid–deletion strain combinations showed significant negative (synergistic) epistasis, the majority of plasmid–mutant combinations (45/54) showed no epistasis or even positive epistasis. Moreover, plasmid costs did not correlate with ribosomal availability, indicating that translational demand is not a major contributor to plasmid-associated fitness costs. Although the bioenergetic costs of plasmid-associated protein synthesis might be large enough to be perceived by selection [34], our results suggest that the fitness effects associated with translation of plasmid genes are negligible compared to other sources of plasmid costs.

This result might seem at odds with previous experimental work that found that translation of foreign proteins is costly, particularly if codon usage greatly differs from that of the host cell [10,15,20,21,35,36]. However, these studies used inducible synthetic systems that overexpressed a test protein (e.g. GFP, LacZ), and the fitness effects detected were only apparent when the overexpressed protein comprised a sizable fraction of the total protein pool of the cell (typically above 5%) [15,20,21,36]. This might be a key difference with our approximation, because despite the relatively high level of expression of plasmid-borne genes [37,38], the overall proportion of plasmid transcripts is relatively low (1–3% of total mRNA levels) [37,39]. If we assume protein levels to be roughly proportional to mRNA levels [40], this result would indicate that plasmid proteins comprise a relatively small fraction of the total protein cellular content, and that translation of plasmid genes is low enough to not significantly disturb the translational machinery of the cell.

The notion that translation is not a major barrier to plasmid spread is supported by several lines of evidence. First, experimental determination of the selective barriers to HGT showed that codon usage is not a good predictor of the fitness effects of transferred genes [41]. Second, the CAI (a measure of codon usage similarity [23]) of plasmid genes weighted by their expression levels does not correlate with plasmid fitness costs [37]. And third, several studies have mechanistically explored the basis of plasmid costs [37,38,42–45], with only one pointing out a causal relationship between translation and plasmid costs [42]. Instead, the key insight that emerges from these studies is that plasmid costs are mainly caused by genomic and metabolic conflicts arising between plasmid and chromosomal genes [37,38,43–45].

It is possible that a reduction of the cellular translation capability will lead to a decrease in plasmid-derived protein levels, thereby relieving the cell from the burden caused by their interaction with cellular networks, and reducing plasmid-associated costs. In addition, the fact that plasmids generally show suboptimal codon usage preferences may further reduce plasmid-derived protein levels, as translation of plasmid proteins is likely to be slower than that of chromosomal proteins. This might mechanistically explain why we (and others [29]) found positive epistasis between *rpsL* mutations and plasmid carriage. In agreement with this idea, plasmid costs are often reduced by mutations in global regulators that lower plasmid gene expression [42]. Our results might also help to explain the observation that plasmids tend to produce smaller costs when cells are growing slowly, such as in poor nutrient conditions [37]. During slow growth, available ribosomal concentrations and translation elongation rates are reduced to better allocate resources [46]. This reduction of the translational capability might contribute towards reducing plasmid-derived protein cellular levels, potentially reducing plasmid-associated genomic conflicts.

However, the effect of *rpsL* and *rrn* mutations in translation is not constrained to plasmid proteins: chromosomal protein levels are also likely to be highly affected, with pleiotropic consequences that are hard to predict [47]. For instance, *rpsL* mutations have been shown to alter complex phenotypes such as virulence or entry into stationary phase [48,49]. We deliberately used two sets of mutants and six diverse plasmids to minimize the biases caused by the pleiotropic effects that these mutants might cause, as they are highly unlikely to equally affect all plasmid–mutant combinations. However, we acknowledge that, given the highly intertwined nature of cellular processes, collateral effects caused by altered translation in *rpsL* mutants or *rrn* deletion strains such as changes in plasmid copy number cannot be ruled out.

A possible limitation of our study is that we measured plasmid costs using established plasmid-bearing clones selected 24 h after conjugation/transformation of the plasmids. However, because many plasmid promoters are controlled by plasmid-encoded repressors, plasmid genes experience a transient transcriptional burst immediately after plasmid acquisition that lasts until plasmid repressors are built up in the recipient cell [50]. This transcriptional overshoot may overwhelm the translational machinery of the cell, transiently increasing plasmid burden upon plasmid reception [51]. Indeed, plasmid acquisition costs have been demonstrated for different conjugative plasmid–bacteria associations [52], suggesting that the translational demand imposed by plasmids immediately after their acquisition may play a key role in long-term plasmid persistence.

Protein translation is arguably the most energetically expensive cellular processes. It has been estimated that protein synthesis accounts for up to 2/3 of cellular ATP consumption and 70% of *E. coli*'s overall resources [53]. These figures evidence that protein synthesis is crucial to ensure the immense ecological success that bacteria have experienced since the origins of life. It is thus likely that plasmid–bacteria coevolution has led to a significant reduction in the translational burden that plasmids might cause [54,55]. New studies aimed at directly quantifying the short- and long-term translational demand of plasmids using large-scale proteomic techniques, as well as direct measures of plasmid-driven ribosomal occupancy via ribosomal profiling experiments, will be needed in order to completely understand the contribution of translational demand to plasmid-associated fitness costs.

## 4. Methods

### (a) Bacterial strains and growth conditions

*Escherichia coli* K-12 MG1655 parental strain and its mutant derivatives were routinely grown in liquid Lennox lysogeny broth (LB, CONDA) with continuous shaking (225 r.p.m.) or LB agar (15 g l^−1^, CONDA) at 37°C unless indicated. Spontaneous streptomycin-resistant mutants were obtained by plating overnight cultures of MG1655 into LB plates containing streptomycin (100 mg l^−1^; Sigma Aldrich). After 48 h of growth, several resistant colonies were isolated and their *rpsL* genes PCR amplified and Sanger sequenced using the oligos *rpsL*-F (5′-TTGACACCTTTTCGGCATC) and *rpsL*-R (5′-TTAAGCCTTAGGACGCTTCA). Mutant clones carrying the mutations K43T, K43N and K43R were further selected and stored as glycerol stocks. Sequential markerless deletions of the *rrn* operons were performed by Quan *et al.* [31] using the recombineering method [56]. Following Levin *et al.* [32] , we adapted the original strain designation to reflect the number of *rrn* operons deleted as follows: D1 (original designation SQ37), D2 (SQ40), D3 (SQ49), D4 (SQ78), D5 (SQ88) and D6 (SQ110) (electronic supplementary material, figure S5). *rrn* operons are interspersed with a number of tRNA genes, and thus deletion of more than five operons is not viable unless the missing tRNA genes are provided *in trans* [31,57]. Consequently, D5 and D6 strains carry the medium copy plasmid pTRNA67, which complements the required tRNA genes [31,58]. The remaining four deletion strains and parental MG1655 were also transformed with the plasmid pTRNA67 to minimize differences in tRNA availability between strains.

### (b) Plasmids

Plasmids pKAZ3 (accession number KR827392.1), pKP-M1144 (acc. no. KF745070.2), pCEMR (acc. no. MT720903), pOXA-48_K8 (acc. no. MT441554) and pCF12 (acc. no. MT720906) were previously published [59–64]. For simplicity, we refer to pOXA-48_K8 as pOXA-48 throughout the text, as it has been shown to be a pOXA-48-like plasmid [60]. Plasmid pKAZ3, pOXA-48 and pCF12 were conjugated to MG1655 and its mutant derivatives using *E. coli β*3914 as donor strain, which is auxotrophic for diaminopimelic acid [65]. Donor and recipient strains were grown overnight, and 1 ml of each culture was centrifuged 5 min at 1500G. Pelleted cells were resuspended in 100 µl of LB, mixed 1 : 1 and spotted onto LB plates. After 2 h at 37°C, spots were resuspended in saline and appropriate dilutions were plated in selective plates containing either carbenicillin (100 mg l^−1^, NZYTECH) for plasmids pKAZ3 and pOXA-48 or aztreonam (25 mg l^−1^, Bristol-Myers Squibb) for plasmid pCF12. Plasmids pKP-M1144, pCEMR and pKA2Q were purified using a commercial mini-prep kit (Macherey-Nagel), transformed into TSS competent cells [66] and selected on carbenicillin-containing plates. Negative controls were routinely used in transformation and conjugation assays to check for contamination. Plasmid presence was verified by assessing that the antibiotic resistance profile of the strain matched the predicted resistance conferred by the plasmid (electronic supplementary material, table S1).

### (c) GFP production

To measure the effect of *rpsL* mutations and *rrn* deletions on translation efficiency, we measured the production of GFP using flow cytometry. Strains were transformed with plasmid pBGT-1 [28], a plasmid carrying an arabinose-inducible *gfp* gene, and selected on LB plates with carbenicillin. Overnight cultures of pBGT-1 transformed cells were diluted 1 : 10 000 into LB containing 0.5% l-arabinose (Sigma-Aldrich). After 2 h of static incubation at 37°C, GFP fluorescence was measured using a CytoFLEX Flow Cytometer (Beckman Coulter Life Sciences) using custom gating protocols and registering 10 000 events.

### (d) Competition assays

We performed competition assays to measure the relative fitness of plasmid-carrying and plasmid-free strains relative to a standard competitor using flow cytometry as previously reported [67]. In short, all strains were competed against a MG1655 derivative carrying an arabinose-inducible chromosomal copy of *gfp* (MG1655::*gfp*). It is important to note that in the GFP production experiments we measured GFP production from a plasmid, whereas in competition experiments, GFP is produced from the chromosome, and that in both cases, GFP is not produced until arabinose is added to the culture. Pre-cultures were incubated at 37°C with 225 r.p.m. shaking overnight in 96-well plates carrying 200 µl of LB broth per well. Pre-cultures were mixed at 1 : 1 proportion and diluted 1 : 400 in fresh media. Initial proportions of GFP and non-fluorescent competitors were confirmed in a CytoFLEX Platform (Beckman Coulter Life Sciences) flow cytometer, recording 10 000 events per sample. To measure these proportions, we incubated a culture aliquot in NaCl 0.9% containing 0.5% l-arabinose for 1.5 h to induce the expression of the chromosomal GFP. Mixtures were competed for 22 h in LB medium at 37°C with shaking (225 r.p.m.). Final proportions were estimated again by flow cytometry as described above. The fitness of each strain relative to MG1655::*gfp* was calculated using the formula:

$$W=\frac{\log\;(N_{\mathrm{final},gfp-}/N_{\mathrm{initial},gfp-})}{\log\;(N_{\mathrm{final},gfp+}/N_{\mathrm{initial},gfp+})},$$

where *W* is the relative fitness of the non GFP-tagged strain, *N*_initial,*gfp*−_ and *N*_final*,gfp*−_ are the numbers of non GFP-tagged cells before and after the competition and *N*_initial,*gfp+*_ and *N*_final,*gfp+*_ are the numbers of MG1655::*gfp* cells before and after the competition. To account for the possible cost of *gfp* insertion and/or its expression, plasmid-free MG1655 was competed against MG1655::*gfp* and the data were normalized by dividing the relative fitness of plasmid–mutant combinations by the relative fitness obtained for plasmid-free MG1655 (MG1655 pTRNA67 when measuring fitness of the *rrn* deletion strains and their plasmid-carrying derivatives). Specifically, the median result of six independent replicates of the competition MG1655::*gfp* versus plasmid–mutant combination was divided by the median result of six independent replicates of the competition MG1655::*gfp* versus plasmid-free MG1655 (MG1655 pTRNA67 for the competitions involving *rrn* deletion strains), resulting in the fitness value of each strain relative to plasmid-free MG1655. To facilitate comparison across plasmids and genotypes, in figures 2*d* and 3*d* we further normalized these data by dividing the fitness values of each plasmid–mutant combination by the fitness of plasmid-carrying MG1655.

### (e) Data analysis

Data were analysed using custom scripts in R statistical programming software. Epistasis was calculated using the formula *ε* = *W*_(plasmid;mutation)_ − *W*_(plasmid;−)_*W*_(−;mutation)_, where *W*_(plasmid;mutation)_ is the fitness of a given plasmid–mutant combination, *W*_(plasmid;−)_ is the median fitness of the plasmid-carrying wild-type MG1655, and *W*_(−;mutation)_ is the median fitness obtained for a plasmid-free mutant (carrying either *rpsL* mutations or a number of *rrn* deletions) [29]. Therefore, positive epistasis indicates that a given plasmid–mutant combination has a higher fitness than the expected sum of costs of the plasmid and the mutation. Similarly, negative epistasis would indicate that the fitness of the plasmid–mutant combination is lower than that predicted from the fitness calculated for the mutant and the plasmid independently. Epistasis was calculated for all plasmids and strains combinations including the plasmid-free wild-type strain, whose median epistasis equals zero by definition. The results were statistically assessed by comparing the replicate values obtained for each plasmid–mutant combination against those obtained of the plasmid-free wild-type strain using ANOVA followed by Tukey test. This method provided comparable results to those obtained using the error propagation method [29], with the advantage of providing a *p*-value corrected for multiple comparisons. To test for sign epistasis, we used an ANOVA followed by Tukey test to assess if the fitness of a given plasmid–mutant combination was higher than the fitness of either the corresponding plasmid-free mutant or the wild-type MG1655 carrying the plasmid.

### (f) Plasmid analysis

Plasmid sequences were retrieved from Genbank and annotated using Prokka (v. 1.14.6) [68] in combination with RAST algorithm [69]. Per gene CAI was calculated using the EMBOSS package (v. EMBOSS:6.6.0.0), with *E. coli* K-12 codon usage table as a reference (Eecoli.cut). The ratio of plasmid sequencing depth to the average sequencing depth for the chromosome was used as a proxy for plasmid copy number, as previously described [70,71]. Briefly, we mapped the reads of each whole-genome sequence with the specific reference sequence (plasmid or chromosome) and obtained the sequencing depth for the plasmids and the chromosome using SAMtools v. 1.12 [72].

### (g) Whole-genome sequencing of MG1655 mutants and analysis of sequence data

MG1655 wild-type and the mutant strains carrying sequential deletions from one to six out of the seven *E. coli rrn* operons were grown in LB medium at 37°C. Genomic DNA of the seven strains was isolated using the Wizard genomic DNA purification kit (Promega, Madison, WI, USA), following manufacturer's instructions. Whole-genome sequencing was conducted at the Wellcome Trust Centre for Human Genetics (Oxford, UK), using the Illumina HiSeq4000 platform with 125 base pair (bp) paired-end reads for all isolates. Illumina HiSeq4000 technology provided a high coverage (greater than 100×). Trimmomatic v. 0.3348 [73] was used to trim the Illumina sequence reads. SPAdes v. 3.9.049 [74] was used to generate de novo assemblies from the trimmed Illumina sequence reads with the –cov-cut-off flag set to ‘auto’, and additional rounds of Pilon were performed following assembly [75]. QUAST v. 4.6.052 [76] was used to generate assembly statistics. All the de novo assemblies reached enough quality including total size of 4.5 Mb. The total number of contigs over 1 kb was lower than 100 and more than 99% of the assembly comprised contigs greater than 1 kb. Prokka v. 1.554 [68] was used to annotate the de novo assemblies with predicted genes. The specific ‘scars’ remaining upon *rrn* deletion and previously described by Quan *et al.* [31] were identified using BLASTN (v. 2.10.0) and mapped against MG1655 using the BRIG (Blast Ring Image Generator) tool [77].

## Data accessibility

The datasets supporting this article have been uploaded as part of the electronic supplementary material [78].
